# Cancellous osteoma of the coronoid process: a literature review and rare case report

**DOI:** 10.22514/jofph.2024.021

**Published:** 2024-06-12

**Authors:** Zygimantas Petronis, Audra Janovskiene, Marijus Leketas

**Affiliations:** Department of Maxillofacial Surgery, Lithuanian University of Health Sciences, LT-50161 Kaunas, Lithuania

**Keywords:** Osteoma, Benign tumor, Mandible, Mouth opening, Case report

## Abstract

Osteoma is a rare benign tumor primarily affecting the craniofacial skeleton. Coronary osteomas in the coronoid process are uncommon and asymptomatic until they affect mandibular function. This report presents a case of coronoid osteoma with its diagnosis, treatment and surgical approach. Osteoma is a benign tumor composed of well-differentiated bone tissue, with different origins: central, peripheral and extraskeletal. Mandibular coronoid osteomas are rare but important to consider in symptomatic patients. Mandibular osteoma frequency ranges from 22.8% to 81.3%, with a reported frequency of 15.28% in the maxilla. A 63-year-old female presented with facial deformation, limited mouth opening, and associated symptoms, such as intermittent dizziness and nasal congestion. Imaging revealed a well-defined radiopaque lesion in the coronoid process, displacing surrounding structures. Diagnosis confirmed coronoid osteoma. Surgical removal resulted in satisfactory recovery and improved mouth opening. Coronoid osteomas are rare, with limited reported cases. Osteomas are more prevalent in the mandible body than in other locations. Radiographic imaging and histopathological examination are crucial for diagnosis. Radiographic and histological features distinguish osteomas from other lesions. Etiology remains uncertain, with trauma, temporal muscle hyperactivity, or post-traumatic fibrosis as potential causes. Differential diagnosis involves distinguishing osteoma from osteochondroma, osteoblastoma, exostoses and osteosarcoma. Continued research and reporting are necessary to enhance understanding and management of this rare condition.

## 1. Introduction

Osteoma is a benign osteogenic tumor composed of well-differentiated mature bone tissue, ranging in size from slight thickening to large masses [1, 2]. Osteomas are basically craniofacial skeleton bone tumors, that rarely affect other bones in the body. The difference in the origin of osteoma divides them into three types: central—from the endosteum, peripheral—from the periosteum, and extraskeletal usually from muscles [3]. Coronary osteomas are extremely uncommon and slow-growing tumors, usually asymptomatic until their size and position interfere with function which can limit mandibular movement [4, 5]. Osteomas are so rare that they occur in 0.01–0.04% of the population [6]. For this reason, there have been very few described case reports over the years, and in the past 10 years there have been only 3 publications. This report describes one of the rare coronoid osteoma cases, differential diagnosis, treatment and surgical approach.

## 2. Review of the literature

### 2.1 Definition

Osteoma, a small noncancerous growth, is characterized as a benign tumor consisting of highly vascularized connective tissue containing deposited osteoid and trabeculae. This tumor exhibits a tendency to proliferate, resulting in reduced mobility and impaired functioning of the mandible [7].

### 2.2 Frequency

Osteoma of the mandibular coronoid process is a rare occurrence in the field of oral and maxillofacial pathology. Despite its low frequency, it is crucial for dental and medical professionals to be aware of this condition and consider it as a differential diagnosis in patients presenting with related symptoms. The frequency of osteomas in the mandible varied across the studies, ranging from 22.8% to 81.3%. Additionally, the studies reported a frequency of 15.28% for osteomas in the maxilla [8].

### 2.3 Reports

A total of 9 publications describing cases of osteomas were found [5, 9, 10, 11, 12, 13, 14, 15, 16] (Table 1). Given that osteoma growth impairs mandibular movement, 7 studies investigated mouth opening before and after osteoma removal [5, 9, 11, 12, 13, 14, 15, 16]. In the past, X-ray imaging was utilized to diagnose and assess osteoma, whereas more recent research conducted in 1987 employed computer tomography for the same purposes. Among the case reports, the prevailing symptoms frequently noted included trismus (lockjaw), swelling (edema), and a zygomatic arch fracture. Regarding the sizes of the specimens, da Costa Araujo *et al*. [16] documented the largest observed osteoma, measuring 3.5 × 4 × 2.5 cm, while Lewars *et al*. [9] reported the smallest osteoma, which had dimensions of 3 × 1.25 × 1.5 cm. However, it is worth noting that two studies did not provide information regarding the size of the osteomas in their reports [11, 12].

**Table 1.** Mandibular coronoid osteoma cases presented in the literature.

No.	Author, years	Gender, age	Affected side	Symptoms	Mouth opening before	Mouth opening after	Image modeling	Surgery type	Tumor dimensions
1	Lewars, 1959 [9]	Male, 15	R	Lockjaw, edema in the zygomatic region	3	12	X ray Tomography	Extraoral	3 × 1.25 × 1.5 5.625
2	Ord *et al*. [10], 1983	Female, 40	L	lockjaw, edema, paresthesia, fracture of the zygomatic arch	-	-	X-ray	Extraoral	4 × 3.5 × 2 28
3	Plezia, 1984 [11]	Female, 26	R	lockjaw, edema	-	-	X-ray	Intraoral	-
4	Wesley *et al*. [12], 1987	Female, 12	B	Garden’s syndrome	12	32	X-ray Tomography	-	-
5	Kurita *et al*. [13], 1991	Female, 40	R	lockjaw, edema, fracture of the zygomatic arch	17	30	Tomography	Extraoral	5 × 3 × 2 30
6	Chen *et al*. [14], 1998	Female, 28	R	lockjaw	11	28	X-ray Tomography	Extraoral	3 × 2 × 1.5 9
7	Vashishth *et al*. [15], 2013	Female, 26	-	Lockjaw, chewing difficulty, deviation on opening to the right side	20	35	X-ray Tomography	Intraoral	2.5 × 3 × 3 22.5
8	da Costa Araújo *et al*. [16], 2013	Female, 45	R	Lockjaw, Fracture of zygomatic arch, edema	8	25	Tomography	Extraoral Intraoral	3.5 × 4 × 2.5 35
9	Saikrishna D* et al*. [5], 2021	Male, 40	L	Lockjaw	30	45	Tomography	Extraoral Intraoral	4 × 3 × 2 24

*R: right; L: left; B: bilateral.*

## 3. Case report

A 63-year-old female presented at the Oral and Maxillofacial Surgery Department of the Hospital of Lithuanian University of Health Sciences Kaunas Clinics, complaining of a facial deformation on the right side, buccally. The patient also complained for almost a year of being unable to open her mouth adequately, as well as intermittent dizziness and nasal congestion. There was no prior history of concomitant infections, surgeries or maxillofacial trauma. Extraoral examination revealed a firm mass under the zygomatic arch, which was not apparent during the intraoral examination. The patient was unable to make protrusive or lateral movements due to a limited range of motion on the affected side, in contrast to the normal range of motion observed on the healthy side. There were no signs of pain noted. Three-dimensional (3D) reconstruction of the Computed Tomography (CT) images showed a well-defined radiopaque lesion of the size of 4.0 × 4.1 × 3.9 (in cm; Fig. 1) with intraosseous and parosteal involvement, expansively dislocating surrounding structures, and broadly adhering to the extracranial part of the middle fossa of the skull base and moderately deforming the inferior part of the greater wing of the sphenoid bone (Fig. 1). On the right side, the mandible was positioned caudally and the mandibular condyle—antero-caudally. M. pterygoideus lateralis was dislocated medially. Dislocation of the mandibular nerve (CN V3) was reported to be also probable. On the left, the jaw was in its normal position.

**Fig. 1.** CT images showing radiopaque lesion. (A) view on an axial plane; (B) view on a coronal plane; (C) view on a sagittal plane.

The angiography of the neck vessels showed no direct contact between the mass and carotid artery or jugular vein, although a. maxillaris and its branches were visible in a caudolateral position, compressed near the formation.

Based on the clinical and imaging features and the slow progressive nature, a preliminary diagnosis of a benign osseous neoplasm of the coronoid process of the right side was established.

Under general anesthesia, using an intraoral approach, the incision was made in the retromolar area on the right side, soft tissues were bluntly dissected, osteotomy of the coronoid process and tumor mass was performed, and the surgical specimen was sent for histopathological examination (Fig. 2).

**Fig. 2.** Histopathological Microscopic View of osteoma. (a) Osteoma histopathological microscopic view; (b) Another site of osteoma histopathological microscopic view.

The specimen measured about 4 cm at its largest diameter and had a nodular surface (Fig. 3). The microscopic analysis revealed a well-circumscribed formation of compact bone tissue with well-vascularised Haversian canals, allowing the diagnosis of osteoma of the coronoid process.

**Fig. 3.** Osteoma with mandible branch and coronoid process.

Recovery following surgery was satisfactory, and the dizziness disappeared. The mouth opening had increased to 32 mm 6 months after, and no occlusal alterations had been noticed. The DC/TMD (Diagnostic Criteria for Temporomandibular Disorders) protocol was employed to assess the degree of mouth opening limitation both before and after the surgical intervention. The preoperative evaluation yielded a score of 81, which subsequently improved to 54 following the surgery [17].

## 4. Discussion

Through a literature review, it was observed that coronoid osteomas are infrequent. The first documented instance of a coronoid osteoma was reported in 1959 by Lewars *et al*. [10]. Out of a total of 87 recorded cases of osteomas, only 9 cases corresponded to osteomas located in the coronoid process. Among all osteomas, the highest prevalence was observed in the body of the mandible, accounting for 41.3% of the total cases. The condyle exhibited a prevalence of 21.83%, while the angle, ramus and coronoid process accounted for 16.09%, 11% and 8% respectively of all mandibular osteomas present in the study [6]. The largest recorded coronoid process osteoma was documented by da Costa Araujo *et al*. [16]. They reported the observation of an osteoma measuring 3.5 × 4 × 2.5 cm in linear dimensions, corresponding to a volume of 35 cm^3^. However, our case report presents a larger osteoma with a volume of 62.4 cm^3^, which is almost twice the volume.

Typically, the diagnosis of osteomas is established through the examination of radiographic images and histopathological specimens. On radiographic images, a characteristic feature of peripheral osteoma is the identification of an oval, well-defined mass that appears radiopaque. This mass is typically attached to the affected cortical bone by a broad base or pedicle. To precisely assess the tumor’s extent and its relationship with nearby anatomical structures, particularly when considering its removal, CT scan, especially with 3D reconstruction, proves to be valuable [6]. From a histological perspective, osteomas exhibit two distinct variations. The first is known as compact osteoma or ivory osteoma, which is characterized by a dense mass of lamellar bone that appears normal, with limited marrow tissue and occasional haversian system. The second variant is the cancellous osteoma, which consists of trabeculae composed of mature lamellar bone. This variant also contains fatty fibrous marrow with osteoblasts interspersed within it [18].

The etiology of osteomas is still under investigation, and various hypotheses have been proposed. However, none of these hypotheses have been definitively proven to date. The most commonly discussed hypothesis suggests that osteomas may be a result of traumas occurring at an early age or hyperactivity of the temporal muscle. Another hypothesis suggests that osteomas could be caused by traumas or infections that occur later in life [19]. The post-traumatic formation of osteomas is based on the theory that hematoma formation can undergo fibrosis, ultimately leading to the development of chondrocytes [7]. However, it is important to note that trauma injuries have only been reported in the medical history of approximately one-third of osteoma patients [20]. The neoplasm hypothesis is also considered but currently lacks conclusive evidence.

In the diagnostic and therapeutic approach to coronoid process osteoma, a systematic sequence of steps is imperative. Comprehensive assessment begins with a computed tomography (CT) scan to precisely delineate the osteoma’s size, location and its potential impact on function in the affected area. Subsequently, a biopsy is conducted to confirm the histopathological nature of the lesion. Once both visual and histopathological evaluations are available, a judicious decision can be made regarding the necessity of osteoma removal. When the osteoma is small in size and does not adversely affect overall function, a conservative approach involving observation may be warranted. Conversely, in cases where the osteoma is substantial and poses a threat to functional aspects or other critical structures, surgical excision becomes the preferred course of action. The differential diagnosis of osteoma involves distinguishing it from osteochondroma, osteoblastoma, maxillary and mandibular exostoses, and osteosarcoma. Radiographic and histopathological examinations are essential for differentiating osteoma from similar lesions. Osteochondroma can be discerned from osteoma by the presence of a cartilaginous cap. Radiographically, osteoblastoma appears as a lesion with a surrounding capsule, while histopathological analysis reveals multinucleated giant cells and vascularization [21, 22]. Symptoms such as swelling, tenderness, anesthesia or paresthesia may indicate a diagnosis of osteosarcoma or osteogenic sarcoma [23]. Exostoses, like osteomas, are typically asymptomatic and can be differentiated based on their location. Exostoses are commonly found in the lingual regions (torus mandibularis) and hard palate (torus palatinus). Additionally, exostoses cease growing before puberty, in contrast to osteomas, which continue to grow after puberty.

## 5. Conclusion

In conclusion, osteomas are rare benign osteogenic tumors primarily affecting the craniofacial skeleton. Coronoid osteomas, specifically in the coronoid process, are extremely uncommon and often asymptomatic until they interfere with mandibular function. Diagnosis of osteomas is established through radiographic imaging and histopathological examination, while surgical removal is the preferred treatment approach. Despite their rarity, it is important for healthcare professionals to be aware of and consider osteomas in patients presenting with relevant symptoms.

Moreover, based on our case report, we present a larger osteoma with a volume of 62.4 cm^3^, which is almost twice the volume of previously reported cases. Our findings highlight the significance of continued research and reporting of cases to enhance the understanding and management of this rare condition, especially considering that our osteoma was the largest among all represented in the past.

## 6. Highlights

1. Osteomas are rare benign osteogenic tumors primarily found in the craniofacial skeleton.

2. Coronary osteomas in the coronoid process are extremely uncommon and often asymptomatic until they interfere with mandibular function.

3. Diagnosis of osteomas is established through radiographic imaging and histopathological examination.

4. Surgical removal is the primary treatment for osteomas and can lead to significant improvement in symptoms and restoration of mandibular movement.

5. Differential diagnosis of osteomas involves distinguishing them from osteochondroma, osteoblastoma, maxillary and mandibular exostoses, and osteosarcoma through careful radiographic and histopathological analysis.
